# Isolation of exosomes from whole blood by a new microfluidic device: proof of concept application in the diagnosis and monitoring of pancreatic cancer

**DOI:** 10.1186/s12951-020-00701-7

**Published:** 2020-10-22

**Authors:** María Sancho-Albero, Víctor Sebastián, Javier Sesé, Roberto Pazo-Cid, Gracia Mendoza, Manuel Arruebo, Pilar Martín-Duque, Jesús Santamaría

**Affiliations:** 1 Department of Chemical Engineering, University of Zaragoza, 50018 Zaragoza, Spain; 2 Instituto de Nanociencia y Materiales de Aragón (INMA), CSIC-Universidad de Zaragoza, 50009 Zaragoza, Spain; 3 Networking Research Center on Bioengineering, Biomaterials and Nanomedicine, CIBER-BBN, 28029 Madrid, Spain; 4 Department of Condensed Matter Physics, University of Zaragoza, 50009 Zaragoza, Spain; 5 Medical Oncology Service, Miguel Servet Hospital, 50009 Zaragoza, Spain; 6 Instituto de Investigación Sanitaria de Aragón (IIS-Aragón), 50009 Zaragoza, Spain; 7 Health Sciences Institute of Aragón (IACS), 50009 Zaragoza, Spain; 8 Fundación Araid, 50018 Zaragoza, Spain; 9 Universidad San Jorge, 50830 Zaragoza, Spain

**Keywords:** Exosomes, Magnetic capture, Microfluidics and pancreatic cancer

## Abstract

**Background:**

Exosomes are endocytic-extracellular vesicles with a diameter around 100 nm that play an essential role on the communication between cells. In fact, they have been proposed as candidates for the diagnosis and the monitoring of different pathologies (such as Parkinson, Alzheimer, diabetes, cardiac damage, infection diseases or cancer).

**Results:**

In this study, magnetic nanoparticles (Fe_3_O_4_NPs) were successfully functionalized with an exosome-binding antibody (anti-CD9) to mediate the magnetic capture in a microdevice. This was carried out under flow in a 1.6 mm (outer diameter) microchannel whose wall was in contact with a set of NdFeB permanent magnets, giving a high magnetic field across the channel diameter that allowed exosome separation with a high yield. To show the usefulness of the method, the direct capture of exosomes from whole blood of patients with pancreatic cancer (PC) was performed, as a proof of concept. The captured exosomes were then subjected to analysis of CA19-9, a protein often used to monitor PC patients.

**Conclusions:**

Here, we describe a new microfluidic device and the procedure for the isolation of exosomes from whole blood, without any need of previous isolation steps, thereby facilitating translation to the clinic. The results show that, for the cases analyzed, the evaluation of CA19-9 in exosomes was highly sensitive, compared to serum samples.
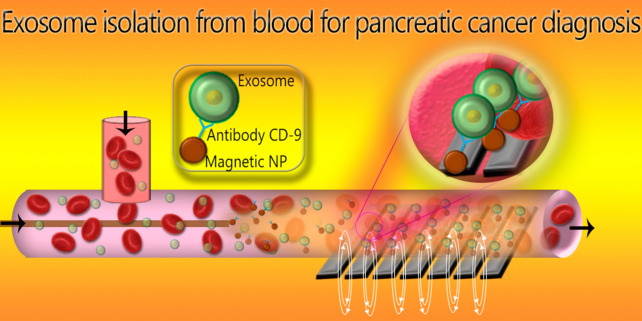

## Background

Exosomes are nano-sized  vesicles with a diameter from approximately 30 to 150 nm secreted by a wide range of cells including immune, neuronal, cancer and stem cells [1, 2]. They are delimited by a cell-type lipidic membrane and, depending on their parental cell and tissue of origin, they are also enriched in specific cargo of proteins and RNAs [3–5]. In fact, some works affirm that the proteome and the transcriptome of exosomes mirror their parental cells, highlighting the employment of this molecular signature in exosomes for the treatment and diagnosis of different pathologies [6–10].

Bioavailability and accessibility in body fluids (blood, semen, breast milk, urine, cerebrospinal fluid and saliva) make of exosomes very useful minimally invasive tools for diagnosis and prognostic applications [11–13]. In fact, they have been proposed as excellent candidates for the diagnostic and the monitoring of Parkinson, Alzheimer, diabetes, cardiac damages, infectious diseases or cancer [14–17].

The most widely employed purification procedure for exosome isolation is differential ultracentrifugation. This strategy is based on the staged removal of whole cells, cellular debris and large organelles based on their different sizes and densities [18]. This technique is laborious, time consuming, expensive and in the case of exosome isolation from whole blood, the purification yields are significantly low, from 5 to 40% [19]. In addition, ultracentrifugation requires high volumes of sample, an obvious limitation regarding to patient blood samples [18]. Although other discontinuous methods have been developed in recent years (immunoaffinity capture, commercial kits, nanostructured based filtration, size exclusion chromatography, acoustic platforms or dialysis membrane filtration), they frequently suffer from limitations including the necessity of additional reagents, long processing time, low reproducibility and low exosome purity [20]. On the contrary, microfluidic devices are now emerging as novel platforms for exosome isolation [21, 22]. These technologies have advantages such as specific separation with high purity, low cost, size uniformity of isolated exosomes and speed of processing compared to other methods [21]. Among the microfluidic-based technologies, the immune affinity approach is the most widely used with antibodies grafted on the vesicles surface (e.g., CD63, CD9 or CD81) that enable specific recognition from other membrane-derived vesicles and lipidic structures. To date, several microfluidic-based techniques have been employed for exosomes analysis [21, 23, 24].

Oncology is perhaps the most promising field of application of exosome-based diagnosis. It has been described that tumor-exosomes reflect the composition of tumor cells, highlighting their potential use in cancer diagnosis. Indeed, tumor cells have been described to secrete a significantly higher amount of extracellular vesicles (EVs) compared to the levels of healthy cells, for instance in metastatic melanoma patients [25]. In fact, Balaj et al. [26], observed that one cancer cell could release from 7000 to 25,000 microvesicles in 48 h whereas a non-pathological fibroblast secretes from 3800 to 6200 vesicles during the same time. Therefore, exosome levels in body fluids from cancer patients (including blood) are significantly higher [27]; and the amount of exosomes secreted by cancer cells (overexpressing some cancer-derived biomarkers) is also higher compared to normal cells. Consequently, the isolation of exosomes from body fluids could provide us with information on a specific pathological condition and gives useful clues about the state or progress of a specific disease. Recent research has focused on circulating exosomes as suitable biomarkers for tumor liquid biopsy [28, 29]. Malla et al. [30], showed that the differential expression of serum exosomal miRNAs before and after radiotherapy can serve as potential indicators of prostate cancer patients’ response to the treatment and prognosis. Furthermore, exosomal proteins are differentially expressed in highly metastatic Panc01-H7 cells versus weakly metastatic Pan01 cells, providing a potential way to classify the aggressiveness of the tumors [31].

Monitoring exosomes also seems to be advantageous versus the monitoring of circulating tumor cells (CTC), since the concentration of exosomes from tumor cells in blood is much higher than the amount of CTCs (1–10 circulating CTC/mL against ≥ 10^9^ vesicles/mL in blood). This abundance could allow the detection and diagnosis of early stage tumors, before a metastatic phase develops [24, 32, 33].

In summary tumor-associated exosomes present significant advantages as potential diagnosis tools for cancer including: (1) their content is related to the cell type of origin. Therefore, specific proteins or RNAs are often enriched in these vesicles, (2) exosome content is protected by the lipidic membrane of the vesicles, (3) they are very stable and can be stored for extended periods of time, (4) the number of exosomes secreted by cancer cells is high, and as a consequence they are abundant in the blood of cancer patients.

In this work we have designed a microfluidic separation device based on magnetic nanoparticles (Fe_3_O_4_NPs) functionalized with antibodies. While this separator might be applied for isolation of exosomes from several patients with different pathologies, we have demonstrated the functionality of the device in the separation of exosomes from patients´ whole blood with pancreatic cancer (PC), a condition where early diagnosis is particularly important: poor prognosis is frequent due to late-phase diagnosis in advanced stages [34]. CA19-9 protein is currently the only FDA-approved test to monitor the tumor response and recurrence of PC patients [35]. However, CA19-9 monitoring is unreliable as a sole marker because high levels are also possible in non-specific diseases such as liver damage or bile duct obstruction, diabetes, benign pancreatic disease and even in asymptomatic patients [36, 37]. Furthermore, some PC patients have normal CA19-9 levels [38, 39], and in this case their concentration does not allow detection of the tumor. Because of these shortcomings, several studies have focused on identifying potential blood-based carriers of CA19-9 as an alternative to the free circulating protein [40–44].

However, as explained above conventional technology for exosome separation from whole blood based on ultracentrifugation cycles [45–47] is limited by contamination and exosome losses. As an alternative, a few studies have used microfluidic systems for PC exosomes isolation. Kanwar et al. [48] reported a platform functionalized with anti-CD63 for on-chip isolation of exosomes from serum obtained from metastatic PC patients. They quantified the exosomes using a fluorescent carbocyanine dye that specifically binds to exosomes. In other study a multichannel nanofluidic system was designed to purify exosomes by an immunomagnetic recognition based on CD9, CD81 and EPCAM. The RNA cargo inside the exosomes was profiled, allowing identification of PC in a blind study [49]. Finally, Taller et al. [50], employed a microfluidic based strategy to analyse exosomal RNA based on surface acoustic wave exosome lysis and ion-exchange nanomembrane sensor.

Here, we present a simple microdevice for exosome isolation, using it as a marker of PC in a proof of concept study. First, exosomes present in PBS, fetal bovine serum (FBS) and in whole blood were isolated by CD9-mediated magnetic capture. It must be remarked that exosome capture was done under continuous flow, with a residence time of 30 s. The exosomal identity was verified by the coexpression of a second exosomal protein CD63 quantified by a CD63 ELISA test. Finally, exosomes from the whole blood of PC patients were captured and the exosomal CA19-9 protein levels were evaluated. The results show the potential of exosome separation and analysis as a marker of PC progression, with significant advantages compared to conventional monitoring methods.

## Results and discussion

### Microdevice design and operation

In general, mixture inside the capillary tubings operating in laminar flow regime conditions (Re < 2000) occurs mainly by diffusion. Under these conditions, the mixing time is calculated by the Fick´s theory according to Eq. (1). However, mixing is not very efficient and both axial and radial concentration profiles arise.

1 $${\text{Mixing time}} = {\text{ dt}}^{{2}} /{\text{4D}}$$

where dt is the tubing diameter and D is the diffusion coefficient.

**Fig. 1:**
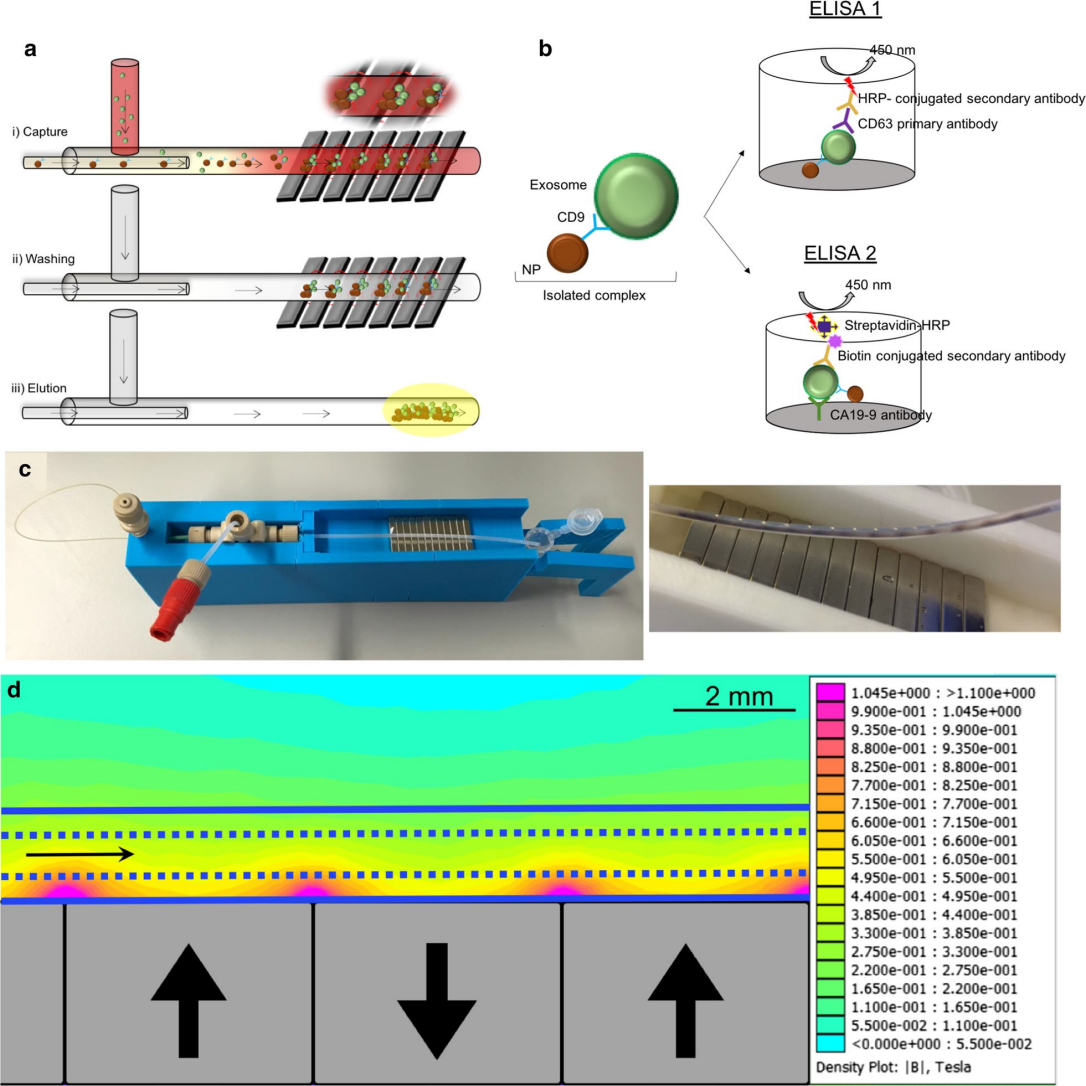
Description of the microdevice. **a** Operation principle of the coaxial mixer. **b** ELISA analysis against CD63 and CA19-9 performed after exosome capture from whole blood. **c** Left: Image of setup. Right: The pipe is lifted from the magnets showing the NPs captured at the junctions between the alternate polarization magnets. **d** Simulation of the magnetic gradient created between each pair of NdFeB magnets. Nanoparticle accumulation coincides with the high intensity nodes. *The tubing is represented with the solid and dashed blue lines*

To face the limited mixing and to promote fast kinetic events (antigen–antibody interaction) in low Re value, a coaxial jet mixer was considered (Fig. 1) [51]. On the one hand, according to previous studies, rapid mixing in coaxial mixers is achieved when the sheath flow is greater than the core flow [51]. Under proper fluid dynamic conditions (shear stress and linear velocity) high velocity streams come into contact with low velocity streams, producing eddy diffusion effects and rapid mixing [52]. On the other hand, the shear stress mismatch between low and fast velocity streams is able to produce turbulence at the point of confluence, promoting entrainment of the low velocity fluid in the fast stream. According to previous results [51], the eddy diffusion effects are generally observed when the sheath flow is significantly faster than the core flow. In this case, a mixing time of 13 s and 55 ms could be just achieved at a total flow rate of 120 μl/min if the ratio core flow/sheath flow were ranged from 1|1 to 1|11, respectively [51].

The operation principle of the coaxial mixer is described in detail in the experimental section and it is schematized in Fig. 1. A series of 12 (4 mm wide) NdFeB magnets were placed in contact with the outer wall of a PTFE duct with an outside diameter of 1.6 mm. The magnetic field intensity was high across the channel, but highest at the junctions between the magnets (Fig. 1d) and thus the Fe_3_O_4_NPs contained in the flowing solution were retained preferentially at these locations. The operating procedure is schematised in Fig. 1a: First, the streams containing patient’s blood and antibody functionalized nanoparticles (Fe_3_O_4_–EDC-NHS-NPs-anti-CD9) were introduced separately in a coaxial mixer and given 30 s to react before arriving to the magnetic separation section. Then, the magnetic nanoparticle-exosome hybrids were trapped by the magnetic action of the permanent magnets and retained in the duct (Fig. 1c right). After capturing the exosome-NPs complexes (Fe_3_O_4_–EDC-NHS-NPs:anti-CD9-Exosomes), these were washed three times with PBS and finally the magnetic complexes were eluted after removing the magnetic field from the device. Once exosomes were purified from whole blood by the recognition of CD9, two different ELISAs were performed to the captured complexes (Fig. 1b): (i) ELISA 1 to estimate the exosomal amount quantified indirectly by CD63 expression and (ii) ELISA 2 to evaluate the amount of CA19-9 protein present in the exosomal sample. The ELISA 1 was carried out to verify that the vesicles captured were indeed exosomes (by the coexpression of CD9 and CD63). And then, once the capture of the exosomes in the microdevice was optimized, blood samples from pancreatic cancer were analyzed to evaluate the CA19-9 present specifically in their exosomes.

Figure 1c shows the experimental setup and the magnetic capture of the exosomes and nanoparticles (NPs) in the channel. A simulation of the magnetic gradient created in the PTFE duct by the NdFeB magnets is depicted in Fig. 1d. The accumulation of magnetic exosome-NPs complexes observed in Fig. 1c is in agreement with the high intensity nodes depicted in Fig. 1d.

### Magnetic nanoparticle synthesis and antibody conjugation

Several works developed antibody-functionalized magnetic nanoparticles in order to develop immunocapture procedures for exosome isolation [21, 23, 48]. Antibody conjugation approaches include covalent coupling to NPs surface and non-covalent interactions, such us host–guest interactions. In the last years, the employment of carboxyl-to-amine crosslinking has been extensively exploited due to its reproducibility and simplicity [53]. One of the main advantages of this procedure is that the antibody binding takes place in buffer and at room temperature.

**Fig. 2:**
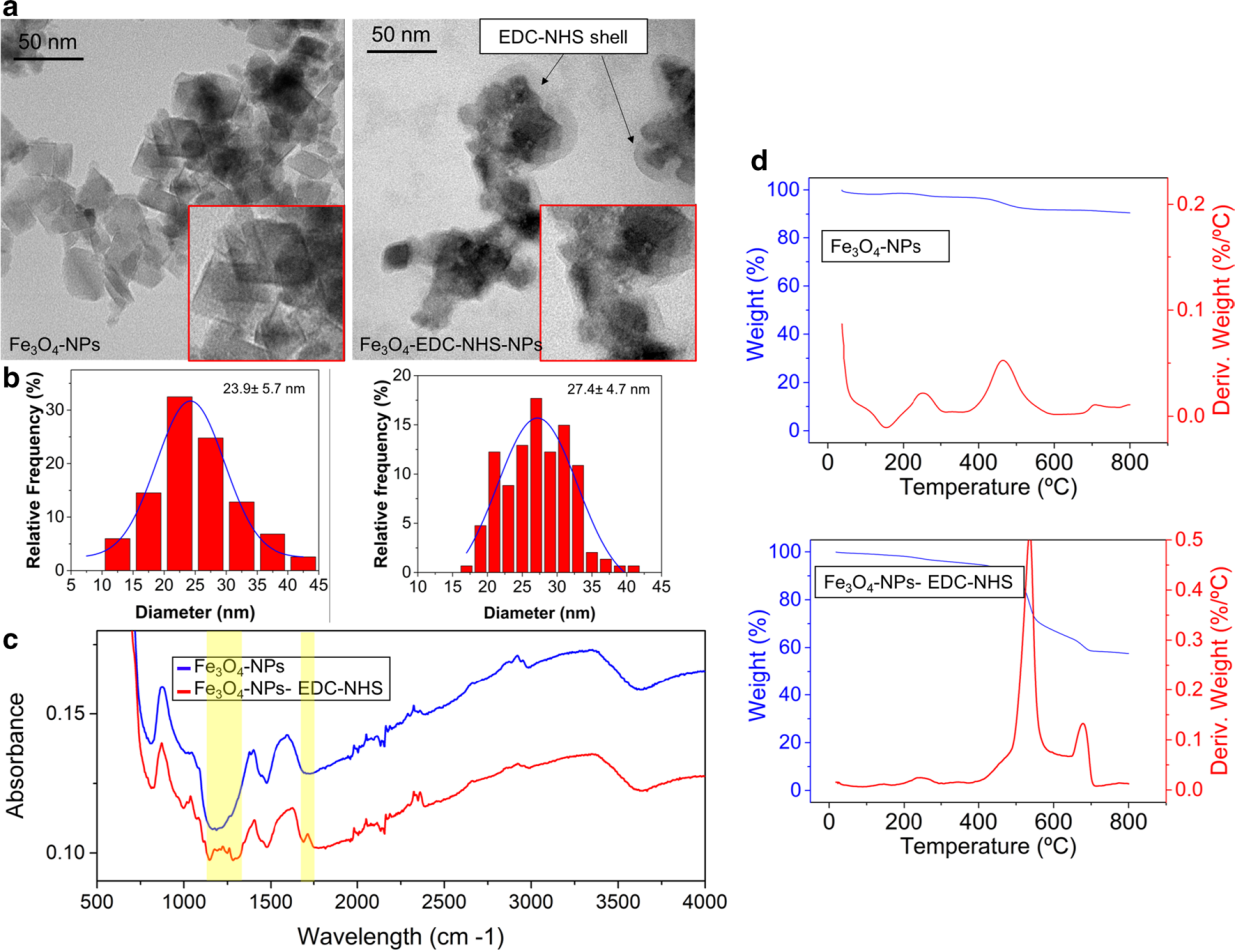
Characterization of Fe_3_O_4_NPs before and after EDC-NHS functionalization. **a** TEM images and size distribution histograms of the naked and the functionalized NPs. The EDC-NHS shell is clearly visible around functionalized NPs. **b** Size distribution histograms of both nanoparticles. **c** FT-IR analysis of the NPs solutions. **d** TGA results of the Fe_3_O_4_NPs before and after EDC-NHS functionalization

As described in the experimental section, after the synthesis, the Fe_3_O_4_NPs with a citrate coating were magnetically separated from the reaction products and washed to remove unreacted precursors. The conjugation of the NPs with the antibody was carried out using a carbodiimide-succinimide covalent linker (see “Experimental” section), to obtain a colloidal dispersion of Fe_3_O_4_–EDC-NHS-NPs. In this reaction, the carboxylic group is first activated by N-ethyl-N’-(3-(dimethylamino)propyl)carbodiimide (EDC) forming an ester, which rapidly reacts with N-hydroxysuccinimide (NHS) [54]. Figure 2a presents representative Transmission Electron Microscopy (TEM) images of both naked (where the term naked denotes magnetite NPs with only a citrate coating) and EDC-NHS grafted nanoparticles. The naked magnetite NPs presented a polyhedral shape with very marked edges whereas the Fe_3_O_4_–EDC-NHS-NPs showed an organic shell around them as well as less sharp edges compared with naked NPs (Fig. 2a). The mean diameter of Fe_3_O_4_NPs measured by TEM was 23.9 ± 5.7 nm whereas the mean diameter of the Fe_3_O_4_–EDC-NHS-NPs was 27.4 ± 4.6 (Fig. 2b). Fourier Transform Infrared (FT-IR) results (Fig. 2c) provide evidence of the functionalization of the NPs with EDC-NHS. FT-IR spectra reveal that two characteristic bands at 1274 cm-1 (corresponding with C-N stretching) [55] and at 1650 cm^−1^ (assigned to the C=O stretching) [56] appeared when the amide bond was formed between the carboxylic group of the citrate group of the Fe_3_O_4_NPs and the amine from the EDC. The amount of EDC and NHS present in the Fe_3_O_4_NPs could be quantified by Thermogravimetric Analysis (TGA). Figure 2d indicates that in the case of the naked NPs the amount of organic material was relatively low (around 5 wt. % of the NPs mass) corresponding with the presence of citrate groups on the NPs surface. On the contrary the TGA analysis of Fe_3_O_4_–EDC-NHS-NPs clearly revealed that the amount of organic material significantly increased to almost 40 wt. % of the total nanoparticle mass, due to the presence of EDC and NHS functionalization. The magnetic properties of Fe_3_O_4_–EDC-NHS-NPs were measured at room temperature using vibrating-sample magnetometer (VSM). The hysteresis loop of the particles is shown in Additional file 1: Figure S1 and shows the expected superparamagnetic properties. This is in agreement with the magnetic properties of naked NPs [57]. Finally, at pH = 7 the zeta potential of the Fe_3_O_4_NPs was − 29.4 ± 1.0 mV, similar to other citrate-coated iron oxide NPs [58], while for and Fe_3_O_4_–EDC-NHS-NPs it was + 16.14 ± 1.04 mV, a strong change that also corroborates functionalization.

**Fig. 3:**
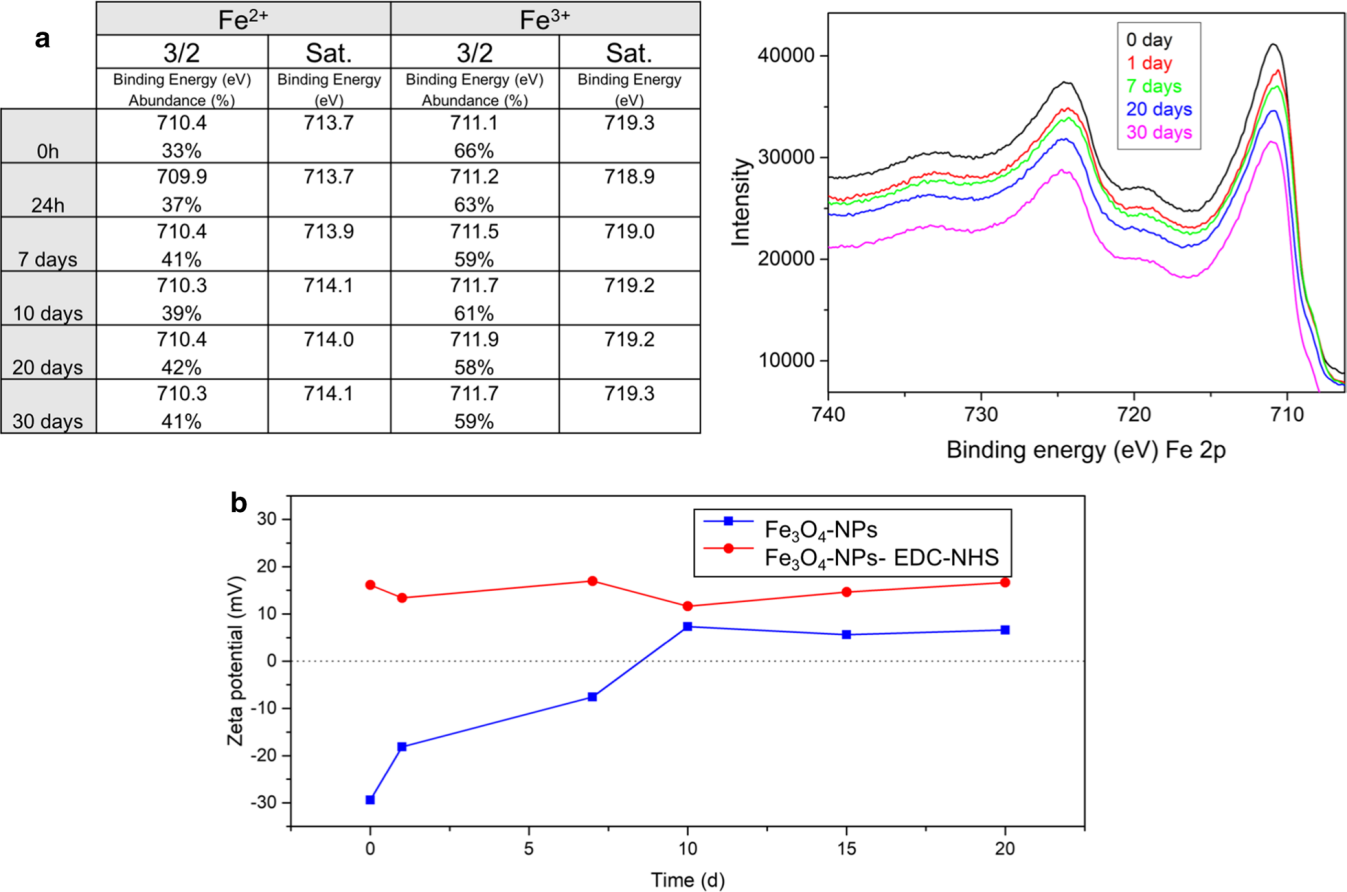
Stability of the magnetic NPs with time. **a** Analysis of Fe oxidation state by XPS in the naked MNPs. **b** Zeta potential evolution of naked and EDC-NHS functionalized NPs

The stability of the Fe_3_O_4_–EDC-NHS-NPs coating was evaluated by X-Ray photoelectron spectroscopy (XPS) and Dynamic Light Scattering (DLS) for 30 days. XPS analysis (Fig. 3a) shows the evolution of NPs composition. For freshly synthesized nanoparticles the typical magnetite ratio (an abundance of 33 at. % and 66 at. % of Fe^2+^and Fe^3+^) is observed. In the binding energy range of 700–740 eV, double peaks with binding energies of Fe 2p3/2 and Fe 2p1/2 at 710.6 and 724.8 eV can be observed, and it is also noteworthy the absence of the satellite peak around 719 eV, which is typical of the maghemite phase [59]. Nevertheless, as time increases, the amount of Fe^2+^ gradually increasing while the Fe_3+_ decreased. We attributed this iron reduction to the presence of the amide bond with the EDC-NHS, thus the nitrogen gradually displaces the Fe^3+^ to Fe^2+^ (Additional file 1: Figure S2A and B) [60]. In the Additional file the evolution of compositions for Fe_3_O_4_NPs and Fe_3_O_4_–EDC-NHS-NPs for 30 days after synthesis is summarized (Additional file 1: Figure S2A). Figure 3b indicates that although the surface charge of the Fe_3_O_4_–EDC-NHS-NPs was unchanged during 20 days (with a slight change around the value of + 15 mV, approximately), the zeta potential of the Fe_3_O_4_NPs changed from negative to positive 7 days after their synthesis, which strongly suggests the loss of part of the citrate stabilization in the naked NPs. Zeta potential analysis of the NPs during 20 days evidenced the stability of the NPs functionalized with the EDC-NHS compared with the naked NPs. TEM images, FT-IR, TGA and Zeta potential results confirmed the functionalization of the magnetite nanoparticles with EDC-NHS for the subsequent antibody binding strategy [61, 62].

### Antibody adherence to coated particles

**Fig. 4:**
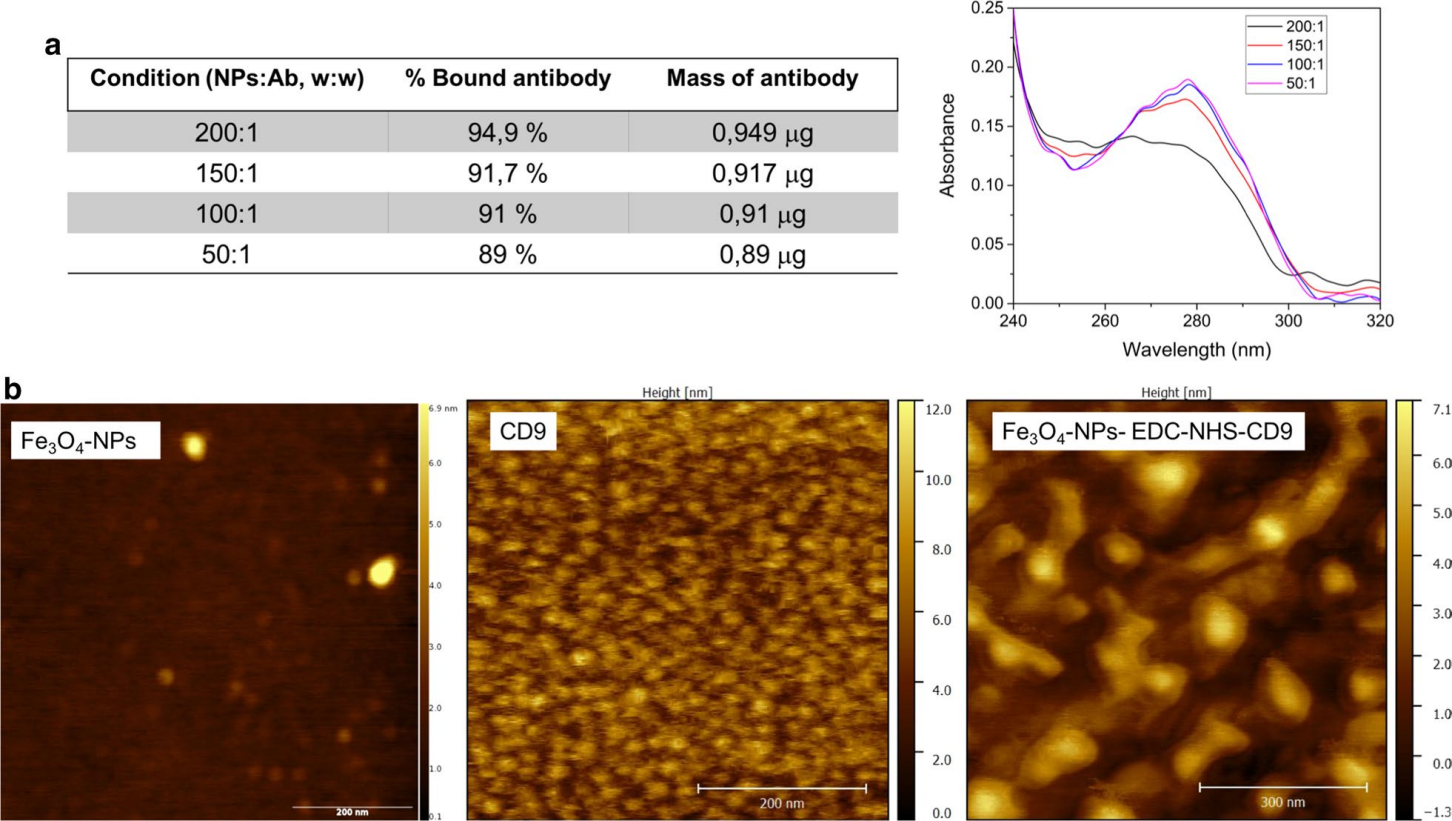
Characterization of antibody coupling. **a** Nanodrop measurements of the NPs-CD9 complexes at different NP/antibody weight ratios. **b** AFM images of the Fe_3_O_4_NPs, of CD9 antibody and of Fe_3_O_4_–EDC-NHS-NPs:anti-CD9

The immobilization of the antibodies on the surface of NPs is a key step in the development of microdevices based on a magnetic capture. Since Fe_3_O_4_–EDC-NHS-NPs present good colloidal stability for at least 20 days, we used them for the covalent bonding of a commercial antibody (anti-CD9) to facilitate the application of this new vector in our microdevice. Nanodrop spectrophotometry measurements at 280 nm clearly indicated that when increasing the NPs:Ab w:w ratio, the amount of free CD9 in the supernatant decreased, demonstrating the efficient binding of the antibody to the Fe_3_O_4_–EDC-NHS-NPs. A high absorbance signal at 280 nm was detected for the lowest amount of nanoparticles (the 50:1 sample) whereas, for a ratio of 200:1 the peak at 280 nm disappeared (Fig. 4a), indicating that the entire antibodies were conjugated to the surface of the particles. Our results indicate that when we increased the amount of NPs, more antibodies were immobilized on the NPs surface. However, Saha et al. [63] concluded that when covering a particle with antibodies not all the antibodies were able to capture the specific antigen. Moreover they evidenced that at a certain point, the number of active antibodies decreases even more when the antibody coverage increases due to steric hindrance.

**Fig. 5:**
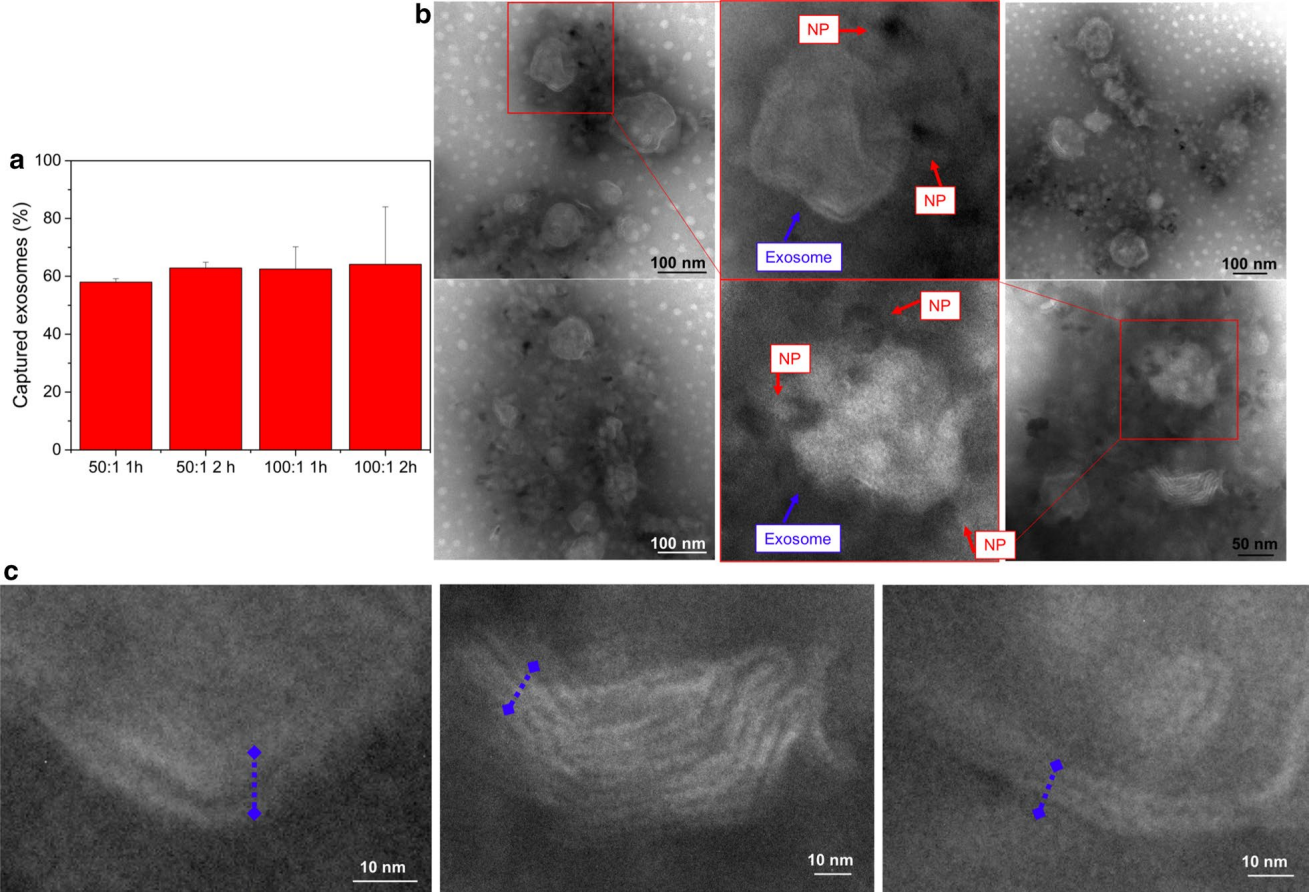
Optimization of exosome capture. **a** Binding efficiency of exosomes by Fe_3_O_4_–EDC-NHS-NPs at different time points at 50:1 and 100:1 (NPs:Ab) weight ratios. **b** TEM images of exosomes captured by Fe_3_O_4_–EDC-NHS-NPs:anti-CD9. **c** High magnification images allow to visualize the double membrane of the exosomes in the complexes

The NPs-CD9 complexes were studied under Atomic Force Microscopy (AFM) (Fig. 4b). According to AFM images, lateral sizes of the Fe_3_O_4_-NPs varied from 30 to 50 nm. Also, observation of CD9 antibody alone on a mica surface showed a homogeneous monolayer of antibodies coating the surface (Fig. 4b). This is in contrast with AFM observations on a mica surface coated with the antibody-functionalized nanoparticles, where distinct features can be observed. In particular, it is possible to observe the binding of the particles (central core in the nanoparticles of almost 7 nm in height, corresponding with the magnetic core) to the CD9 antibody (arms surrounding the NPs of 4–5 nm height, similar to the CD9 alone). Thus, these results confirmed an increase on NPs diameter when they were covered with the CD9, (compared with naked NPs) evidencing the successful coupling of the particles with the antibody. Finally and before attempting to capture exosomes in the microdevice, the functionality of the Fe_3_O_4_-EDC-NHS-NPs: anti-CD9 complexes was tested in bulk. To do that, 40 μg of exosomes were put in contact with the functionalized Fe_3_O_4_NPs surface covered with anti-CD9 at two different NPs:Ab ratios (100:1 and 50:1). Although it seems logical that increasing the NPs:Ab ratio and the time of incubation should lead to a higher exosomes capture, only slight and not statistically significant differences were found (Fig. 5a). Representative TEM images of the exosomes recognized by the Fe_3_O_4_-EDC-NHS-NPs:anti-CD9 complexes are showed in Fig. 5b. Captured exosomes appear bound to an organic shell (attributed to the EDC-NHS and anti-CD9 functionalization) and the Fe_3_O_4_NPs. The characteristic double membrane of exosomes is clearly visualized in the captured exosomes (Fig. 5c). On the contrary, when using the magnetic NPs without the exosomal antibody, no exosomes were captured (results not shown).

### Exosome capture in the microfluidic system

**Fig. 6:**
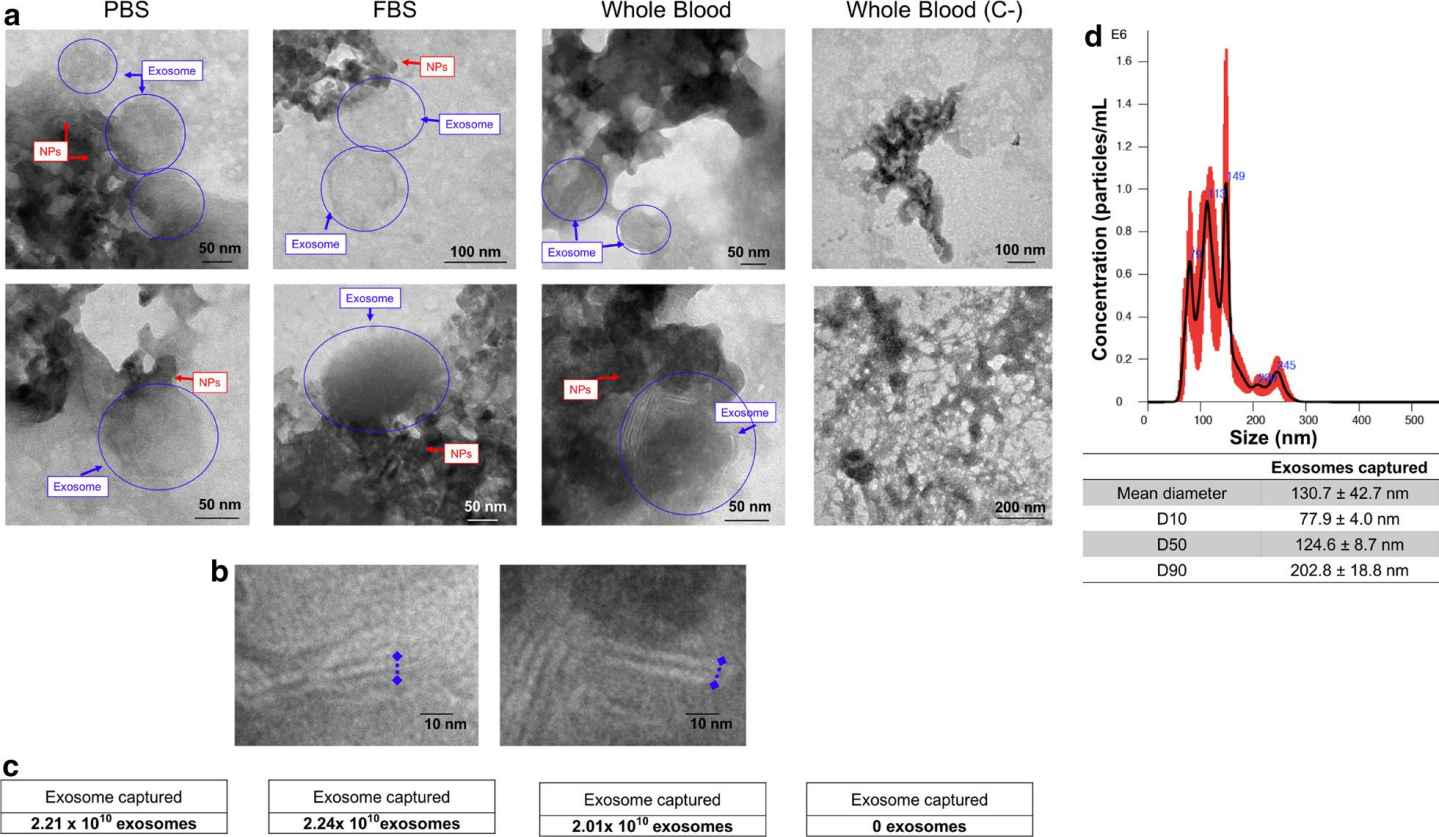
**a** TEM images from different locations of exosomes captured by the microfluidic device from PBS, FBS and whole blood. A negative control is also shown (Fe_3_O_4_NPs-EDC-NHS without the antibody). The blue arrow indicates the membrane of the exosome surrounded by the particles. **b** Exosomes are in indicated with blue circles and arrows whereas the magnetic nanoparticles covering them are signaled with red arrows. **c** High Magnification images to visualize the double membrane of the exosomes in the complexes. ELISA CD63 results indicating the number of exosomes captured. **d** Particle size analysis of the captured exosomes in the microfluidic device by NTA

The capability of the microdevice was tested with exosomes in PBS and FBS. In TEM images of exosomes captured from PBS, FBS and whole blood (Fig. 6a) it is possible to observe the presence of the captured exosomes surrounded by the magnetic nanoparticles. Unavoidable, drying stages resulted in non-uniform nanoparticle deposition and the formation of nanoparticle aggregates located in segregated patches. This phenomena can be explained by the geometric constraints at the contact line of the evaporating droplet during surface dewetting and the so called ‘coffee-ring’ effect [64]. However, the quality of TEM images is high enough to determine the presence of exosome-NPs complexes. In some of these complexes, the double lipid membrane of exosomes is clearly observed (Fig. 6b). Once the exosomes were captured in the microdevice by the formation of the Fe_3_O_4_–EDC-NHS-NPs:anti-CD9 complexes, the amount of CD63 protein present was evaluated by an ELISA test (see experimental section) and the number of captured exosomes was obtained (Fig. 1b). In Fig. 6c the number of exosomes captured from PBS, FBS and whole blood is given. In all the samples, approximately 2 × 10^10^ exosomes were captured when employing only 50 μg of Fe_3_O_4_–EDC-NHS-NPs bound to anti-CD9 at 100:1 ratio in 500 μL. As a negative control, whole blood was injected thought the microdevice in presence of naked Fe_3_O_4_–EDC-NHS-NPs (without antibody functionalization). TEM images and antiCD63 ELISA results demonstrated the absence of exosomes after the capture, confirming the specific capture of exosomes by antibody-functionalized nanoparticles. Finally, Nanoparticle Tracking Analysis (NTA) analysis of the captured exosomes was performed. Figure 6d  reveals a diameter of the particles and exosomes complexes of 130 nm. According to previous studies, exosomes have a diameter ranged from 30 to 100 nm. Additional file 1: Figure S3A  shows a NTA analysis of control exosomes, revealing a mean diameter around 100 nm. On the contrary, the DLS of the particles alone indicate a mean hydrodynamic diameter of approximately 68 nm (Additional file 1: Figure S3B), also in concordance with the previous results considering the intrinsic agglomeration of the particles due to their magnetic properties.

**Fig. 7:**
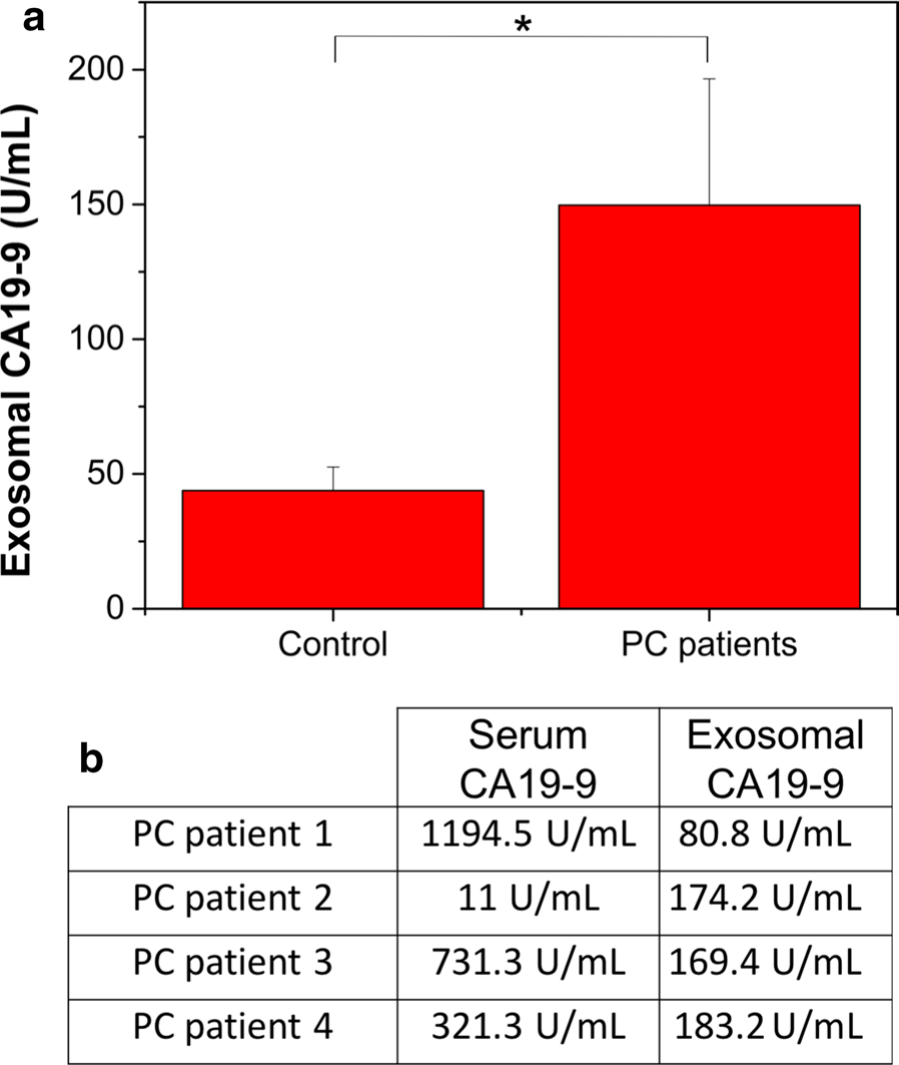
Analysis CA19-9 levels present in whole blood from PC patients. **a** ELISA CA19-9 results indicating the overexpression of the proteins in the exosomes isolated from PC patients compared with healthy donors. **b** Serum CA19-9 levels and exosomal CA19-9 levels in PC patients. The PC patient II represents the false negative case, whose free serum CA19-9 levels were in the normal range whereas the exosomal CA19-9 levels were significantly increased

Once the functionality of the method had been established, the microfluidic device was employed to capture exosomes from whole blood. The possibility of using exosome monitoring as a diagnostic tool was explored by analysing the amount of CA19-9 present in exosomes captured from whole blood. Figure 7a shows that CA19-9 levels in exosomes captured from whole blood were significantly higher in PC patients comparing with the levels of healthy donors. The sensitivity of exosome analysis is highlighted in the results obtained for patient 2, a false negative case considering serum analysis (the free CA19-9 level in serum was normal, its exosomal CA19-9 was significantly higher, giving a valuable diagnostic indication). The content of CA19-9 protein in the captured exosomes was used to identify PC patients from samples from healthy donors. The CA19-9 analysis in the captured exosomes was more sensitive than in serum, opening a window of opportunity to avoid false negatives from the serum analysis, a common problem that limits the applicability of these biomarkers [65]. As Fig. 7b summarizes, serum CA19-9 levels were not elevated (< 37 U/mL) [65] in one of the four evaluated patients, being a false negative. On the contrary, CA19-9 exosomal levels were overexpressed in the four PC subjects analysed. Although some aggregation of exosomes and nanoparticles was observed by TEM images (Fig. 6a), the specific and selective exosome capture was strong enough (Fig. 6b) to detect the CA19-9 exosomal levels with a high sensitivity compared with free serum levels.

Exosomal CA19-9 levels were clearly enriched in all PC patients’ samples analysed compared to those from healthy donors. This is consistent with the results of Javeed et al. [66], who reported that EVs in PC patients were predominantly exosomes and their WB analysis revealed the presence of CA19-9 in all the PC patient-derived exosome samples. They also showed that exosomes from control patients showed no CA19-9 levels. The amount of exosomes in PC patients was significantly higher compared with control group, suggesting that the difference is due to the production by pancreatic tumor cells [66]. However, their procedure involved a laborious series of ultracentrifugation and purification steps, a process that compromises reproducibility. In contrast, the microdevice presented in this work allows to capture exosomes from blood easily even from whole blood samples, facilitating translation to the clinic. Although the proof of concept has been performed with PC patient samples, the microfluidic device could in principle be used for the diagnosis of other pathologies just by changing the antibody of interest.

## Conclusions

The isolation of exosomes and their subsequent analysis is emerging as a promising technique in diagnostics and personalized medicine. However, current methods based on sequential centrifugation steps at high speeds require expensive equipment, highly trained personnel and are not amenable to automatic operation, which compromises reproducibility of analysis. In contrast, the device presented here allows for in-flow capture and separation of exosomes from different fluids, including whole blood. It uses antibody-functionalized magnetic nanoparticles that can be stored prior to use. Rather than a centrifugation/purification cascade, operation involves feeding the sample and the antibody-nanoparticle streams to a coaxial mixer, and collecting the captured exosomes downstream, after allowing for antibody recognition. The procedure is comparatively much faster and the device can be operated by personnel with basic skills. As a proof of concept, the microfluidic platform has been employed for the isolation of PC-derived exosomes from whole blood without the need of any pretreatment, and the results show a high sensitivity of PC-marker detection in the captured exosomes, compared to serum samples. The same operating principle could be adapted to the diagnosis of other diseases where characteristics proteins are also enriched in patient exosomes.

## Methods

### Microdevice fabrication and operation

The inner and outer capillaries of the coaxial mixer were made of Polyether ether ketone-PEEK (hydrophilic) and Polytetrafluoroethylene-PTFE (hydrophobic), respectively. The inner capillary diameter was 150 µm, whereas outer capillary diameter was 350 µm. The functionalized magnetic nanoparticles were injected (core flow) at a flow rate of 10 µL/min, whereas the exosome-containing fluid (sheath flow) was injected at a flow rate of 50 µL/min (core flow: sheath flow ratio 1|5). The flow velocity ratio of the core and sheath streams was 5.5, a value high enough to promote a fast mixing of the functionalized magnetic nanoparticles in the exosome-containing fluid [67]. The inner capillary was coaxially placed under an optical microscope to get an axisymmetric flow-focusing device where the magnetic nanoparticle phase is surrounded symmetrically by the sheath phase. PTFE tubing was selected to avoid surface fouling by blood proteins and other biomolecules. The length of the PTFE tubing (inside diameter of 760 μm) was 10.5 cm (6.5 cm from the inner capillary to the first magnet), an overestimated length to assure a complete mixing [67] and antigen/antibody interaction [68] before magnetic field interrogation. The coaxial capillary system was supported in a polymer housing fabricated by 3D-printing to avoid capillaries misalignment and to favour fluid dynamic reproducibility (Fig. 1c). The support for the device as well as the vial adapter were designed by Autodesk AutoCAD and were also 3D-printed. Two different syringe pumps (Harvard Apparatus PHD ULTRA™) were used to inject core and sheath streams. The coaxial mixer enables the purification of exosomes by inmmunomagnetically binding CD9 (exosomal protein surface marker) directly from unprocessed patients´ whole blood. Finally, a magnetic assembly with NdFeB permanent magnets with alternate polarization was used to achieve a high capture force thanks to the high magnetic field gradient created between each magnet and the contiguous one. The simulation of the magnetic field distribution produced by the assembly of magnets has been performed with the FEMM freeware package (Fig. 1d).

### NPs synthesis and characterization

The citrate functionalized Fe_3_O_4_NPs were synthesized by scaling up a facile one-step method previously developed by Hui et al. [57] All the chemicals were provided by Sigma Aldrich. In brief, 2 mmol of C_6_H_5_Na_3_O_7_·2H_2_O (citric acid, trisodium dehydrate), 8 mmol of NaOH, and 0.4 mol of NaNO_3_ were mixed in 18 mL of distilled H_2_O. Then, the mixture was placed in a three-neck flask and heated to 100 °C. Separately, 2 mL of 2 M FeSO_4_·4H_2_O solution was added into the mixture and it was kept at 100 °C for 1 h, observing a colour change from pale white to dark brown, which corresponds with the formation of the Fe_3_O_4_NPs. The final nanoparticle dispersion was cooled down to room temperature and the Fe_3_O_4_NPs were washed with water by trapping them with a magnet three times. The brown precipitate was finally dispersed in water. Once the Fe_3_O_4_NPs were synthesized, they were functionalized via covalent amide bonding by employing EDC and NHS in order to crosslink the carboxylic acid from the citrate group of the nanoparticles with the primary amines present in the FC region of the antibody (see scheme in Additional file 1). It is well known that the NHS ester is an intermediate product employed for the immobilization of biomolecules (peptides, antibodies, etc.) containing free primary amino groups via amide linkage. First of all, the Fe_3_O_4_NPs were mixed with the EDC (1:1, w:w) for 30 min at room temperature. In order to improve the efficiency of the reaction, the Fe_3_O_4_-EDC-NPs were activated with NHS, forming an NHS ester which is significantly more stable than the intermediate generated between the carboxylic and the EDC groups. Both the EDC and the NHS functionalization of the Fe_3_O_4_NPs were performed at pH 5.5 in buffer MES. Finally, the subsequent Fe_3_O_4_-EDC-NHS-NPs were washed three times with distilled water to stop the crosslinking reaction. Finally, Fe_3_O_4_-EDC-NHS-NPs were conjugated to the primary amines present in the FC region of the antibodies at physiological pH (in Dulbecco’s Phosphate Buffered Saline (DPBS)).

The naked and the EDC-NHS functionalized Fe_3_O_4_NPs were characterized by different physicochemical techniques. First of all, they were thoroughly characterized by TEM operated at 200 kV with a LaB6 electron source fitted with a “SuperTwin®” objective lens. Particle solutions were pipetted onto a TEM copper grid and then they were stained with 3% phosphotungstic acid (Sigma Aldrich, United States). The size distribution of the nanoparticles was obtained employing TEM images using ImageJ software (NIH-RSB) from at least 200 particles. TGA was performed in a temperature range between 30 and 850 °C with a heating rate of 20 °C min^−1^ in N_2_ atmosphere to determine the citrate/Fe as well as the EDC-NHS/Fe ratios in the NPs (Mettler Toledo TGA/STDA 851e). FT-IR spectra were recorded using a Bruker Vertex 70 FTIR spectrometer equipped with a DTGS detector, a horizontal attenuated-total-reflection accessory with thermal control, and a diamond crystal (Golden Gate Heated Single Reflection Diamond ATR, SpectraTecknoroma). The FT-IR spectra both of Fe_3_O_4_NPs and Fe_3_O_4_-EDC-NHS-NPs were collected with 200 scans in the 400–4000 cm^−1^ spectral region at 4 cm^−1^ resolution. Magnetic properties at room temperature were measured by employing a vibrating sample magnetometer (VSM Lake Shore 7410). Finally, the stability of both Fe_3_O_4_NPs and Fe_3_O_4_-EDC-NHS-NPs was evaluated during 30 days by X-ray photoelectron spectroscopy analysis (XPS) and dynamic light scattering DLS. The XPS analysis of the samples was performed with an Axis Ultra DLD (Kratos Tech.) apparatus to determine the relative abundance of Fe_2+_ and Fe_3+_. The samples were collected under glass coverslips and allowed to dry. The spectra were excited by the monochromatized Al Kα source (1486.6 eV) run at 15 kV and 10 mA. For the individual peak regions, a pass energy of 20 eV was employed. Survey spectra were measured at 120 eV pass energy. The CasaXPS sofware was used to analyze the peaks. After the subtraction of the background, a weighted sum of Lorentzian and Gaussian components curve was employed. Furthermore, the zeta potential of both nanoparticle samples was measured at pH = 7 (Brookhaven 90 plus and ZetaPALS software).

### Antibody adherence to coated nanoparticles

Anti-CD9 antibody was purchased from Abcam. Several ratios Fe_3_O_4_-EDC-NHS-NPs:anti-CD9 were investigated in order to optimize the labeling of the nanoparticles with the antibody. The Additional file 1: Figure S4 shows a scheme of the functionalization of the particles. The amount of antibody was kept constant (1 μg) and the NPs mass was varied from 200 to 50 μg (200:1, 150:1, 100:1 and 50:1; w:w) in a final volume of 10 μL (PBS). Thus, 1 μg of antibody was added to the activated NPs and the solutions were incubated for 4 h at room temperature with vortexing every 15 to 30 min. To eliminate the unbound anti-CD9, the Fe_3_O_4_-EDC-NHS-NPs:anti-CD9 were magnetically extracted and washed three times with PBS. To quantify the antibody binding ability, the supernatant of the Fe_3_O_4_-EDC-NHS-NPs:anti-CD9 dispersion was measured with a UV–VIS spectrophotometer (Thermo Scientific Nanodrop 2000) by studying the absorption at 280 nm. Then, the amount of anti-CD9 bounded to the NPs was indirectly estimated from the non-bounded antibody obtained in the supernatant. To estimate the protein concentration in the supernatant, the following formula was employed based on the absorbance of the aromatic rings of the amino acids of proteins (2) [69, 70]:

2 $${\text{Antibody concentration }}\left( {{\text{mg}}/{\text{mL}}} \right) \, = \, ({1}.{55} \cdot {\text{Abs}}_{{{28}0}} {-} \, 0.{76} \cdot {\text{Abs}}_{{{26}0}} )$$

The Fe_3_O_4_-EDC-NHS-NPs:anti-CD9 complexes were visualized following a Quantitative Nanoscale Mechanical characterization (QNM peakforce mapping) by AFM in a Multimode 8 (Bruker Co., Billerica, MA, United States). The tip employed was a commercial tip (SNL model, type—C; Bruker). In order to analyze the anti-CD9 antibody, a protocol previously reported by Ido et al. was followed [71]. Firstly, a 5 µL droplet of a 50 mM MgCl2 solution was deposited onto a freshly cleaved muscovite mica substrate. Then, a 5 µL droplet of the complexes dispersion was dropped on the substrate. Five minutes later, the substrate was gently rinsed five times with dH2O. AFM imaging was performed without drying the sample and the substrate. In order to guarantee that we were observing the Fe_3_O_4_-EDC-NHS-NPs:anti-CD9 complexes, Fe_3_O_4_-NPs, Fe_3_O_4_-EDC-NHS-NPs and CD9 were also analyzed under the AFM.

### Exosome capture

To verify the functionality of the nanoparticles covered with the antibody, the complexes (50:1 and 100:1, NPs:Ab w:w ratio) were incubated at room temperature with 40 µg of exosomes during 1 and 2 h. After that, the amount of captured exosomes was evaluated by indirectly measuring exosomes present in the supernatant. Thus exosomes recognized by the complexes were captured with a magnet and total protein amount of the exosomes present in the supernatant (non-captured exosomes) was quantified by Pierce BCA protein assay (Thermo Fisher Scientific, United States), the standard method for exosome quantification.

### PBS, serum and whole blood preparation

Exosomes present in PBS, serum and total whole blood were captured by the microdevice. In the case of PBS, the suspension was prepared by adding the previously purified exosomes from cell cultures by ultracentrifugation cycles. Exosomes present in FBS were also isolated by the microdevice. Finally, all the assays involving human materials were approved by the Research Ethics Committee of the Community of Aragon (*Comité de Ética de la Investigación de la Comunidad de Aragón -CEICA-): Extracción de exosomas de tumores y plasma de pacientes con diversos grados de malignidad, para el estudio de su composición y correlación clínica, PI18/198*. Participants provided their written informed consent to participate in this study. Three control samples and seven PC patients were employed in this study. All the blood samples were obtained from recently diagnosed stage IV PC patients’ blood samples previously to the initiation of first line therapy and collected in vacutainer sodium citrate tubes in Hospital Universitario Miguel Servet, Zaragoza (Spain). Samples were stored during 2 h at 4 °C until use. 500 μL of blood were employed for the exosomal capture in the microdevice.

### Immunocapture and quantification of exosomes in the microdevice

An image of the experimental setup and the operating procedure together with a scheme of the operation principle are shown in Fig. 1. To capture exosomes, 20 µg of Fe_3_O_4_-EDC-NHS-NPs were conjugated to 200 ng of anti-CD9 in 50 µL of PBS during 4 h. After that, the excess of non-bounded antibody was eliminated and the resulted Fe_3_O_4_-EDC-NHS-NPs:anti-CD9 were dispersed in 500 µL of PBS.

Then, the Fe_3_O_4_-EDC-NHS-NPs:anti-CD9 and the exosome-containing sample were introduced into the microfluidic chip. On the one hand, the magnetic nanoparticles conjugated to the antibody were fed at a flow rate of 10 µL/min in the internal channel. On the other hand, the exosome sample was introduced at a flow rate of 50 µL/min in the external channel. Firstly, exosomes were isolated from three different fluid sources: PBS, FBS and whole blood. As negative control, Fe_3_O_4_-EDC-NHS-NPs without being conjugated to CD9, were also passed through the microdevice in the presence of whole blood. The captured exosomes were finally characterized by TEM and ELISA. For TEM analysis, the captured sample was deposited onto a grid and was fixed with 3% phosphotungstic acid (PTA). They were visualized following the previously described protocol. The number of captured exosomes was quantified by ExoELISA-ULTRA CD63 (System Biosciences, United States). The complexes Fe_3_O_4_-EDC-NHS-NPs:anti-CD9-exosomes captured with the microdevice were deposited onto a 96 well plate and were incubated with an anti-CD63 primary antibody for 1 h. Then a Horseradish Peroxidase (HRP) enzyme linked secondary antibody was employed for signal amplification. A colorimetric substrate of the HRP enzyme was added and the accumulation of the coloured product was measured by a microtiter plate reader at 450 nm absorbance. The accumulation of the coloured product was proportional to the amount of exosomes present in the sample. Finally, the concentration and the diameter of the captured exosomes were measured using Nanosight (Malvern Instruments, UK). With the aim of resuspending homogenously the complexes captured for Nanosight measurements, samples were diluted in PBS and sonicated in a bath after their capture in the magnetic device. Samples were measured at room temperature in triplicate for 60 s.

### Exosome capture: ELISA CA19-9 (blood from PC)

To quantify the exosomal CA19-9 amount present in exosomes of PC blood patients, whole blood (500 μL) was passed through the microdevice and exosomes were isolated as previously mentioned. Then, CA19-9 levels were analyzed by CA19-9 Human ELISA Kit (Thermo Fisher Scientific, United States) following manufacturer instructions. To guarantee that no free CA19-9 protein was captured and to avoid its interferences, the CA19-9 was performed in the previously isolated fraction based on a sandwich capture with anti-CD9 and anti-CD63.

### Statistical analysis

All the results expressed as the mean ± the standard deviation were performed at least in triplicate. The statistical analysis of the data was carried out using the GraphPad Prism 7.04. Significance was determined by one-way analysis of variance (ANOVA): *P < *0.05*.

## Supplementary information

**Additional file 1.** Additional file regarding the complete atomic composition of the nanoparticles and the chemical environment of the C present in the NPs is included. Moreover, in the Additional file it is also shown a scheme of the functionalization process performed with the EDC-NHS linker and the CD9 antibody binding. **Figure S1.**. Hysteresis loop measured at 300 K for the Fe_3_O_4_–EDC-NHS-NPs. The NPs show superparamagnetic properties at room temperature. **Figure S2.**. A) Complete atomic percentage of Fe_3_O_4_NPs and Fe_3_O_4_–EDC-NHS-NPs during 30 days after their synthesis. B) Study of the atomic environment of the C present in the NPs. **Figure S3.**. A) NTA of control exosomes and B) DLS of magnetic nanoparticles employed for the exosome capture. **Figure S4.**. A) Scheme of the functionalization of the MNPs with the EDC-NHS linker, followed by the CD9 antibody binding.

## Abbreviations

NPs: Nanoparticles
Fe_3_O_4_NPs: Magnetic nanoparticles
EDC: *N*-Ethyl *N*’(3(dimethylamino)propyl)carbodiimide
NHS: *N*-Hydroxysuccinimide
Fe_3_O_4_–EDC-NHS-NPs: Magnetic nanoparticles functionalized with EDC and NHS
Fe_3_O_4_–EDC-NHS-NPs: Anti-CD9 antibody
Fe_3_O_4_–EDC-NHS-NPs: Anti-CD9-Exosomes
