# Supplementary material for: Isolation of exosomes from whole blood by a new microfluidic device: proof of concept application in the diagnosis and monitoring of pancreatic cancer
Source: J Nanobiotechnology. 2020 Oct 22;18:150. doi: 10.1186/s12951-020-00701-7 (PMC7579907; doi:10.1186/s12951-020-00701-7)
Supplement: Supplementary file 1 — Additional file 1. Additional file regarding the complete atomic composition of the nanoparticles and the chemical environment of the C present in the NPs is included. Moreover, in the Additional file it is also shown a scheme of the functionalization process performed with the EDC-NHS linker and the CD9 antibody binding. Figure S1.. Hysteresis loop measured at 300 K for the Fe3O4–EDC-NHS-NPs. The NPs show superparamagnetic properties at room temperature. Figure S2.. A) Complete atomic percentage of Fe3O4NPs and Fe3O4–EDC-NHS-NPs during 30 days after their synthesis. B) Study of the atomic environment of the C present in the NPs. Figure S3.. A) NTA of control exosomes and B) DLS of magnetic nanoparticles employed for the exosome capture. Figure S4.. A) Scheme of the functionalization of the MNPs with the EDC-NHS linker, followed by the CD9 antibody binding. [file 12951_2020_701_MOESM1_ESM.docx]

**Additional material**

Isolation of exosomes from whole blood by a new microfluidic device. Proof of concept application in the diagnosis and monitoring of pancreatic cancer.

María Sancho-Albero ^a,b,c^, Víctor Sebastián ^a,b,c,*^, Javier Sesé ^a, d^, Roberto Pazo-Cid ^e^, Gracia Mendoza ^c,f^, Manuel Arruebo ^a,b,c^, Pilar Martín-Duque ^c,f,g,h,*^ and Jesús Santamaría ^a,b,c^

^a^ Department of Chemical Engineering, Aragon Institute of Nanoscience (INA), University of Zaragoza, 5018- Zaragoza, Spain.

^b^ Instituto de Nanociencia y Materiales de Aragón (INMA), CSIC-Universidad de Zaragoza, Zaragoza 50009, Spain.

^c^ Networking Research Center on Bioengineering, biomaterials and Nanomedicine, CIBER-BBN, 28029-Madrid, Spain.

^d^ Department of Condensed Matter Physics, University of Zaragoza, 50009-Zaragoza, Spain.

^e^ Medical Oncology Service, Miguel Servet Hospital, 50009-Zaragoza, Spain.

^f^ Instituto de Investigación Sanitaria de Aragón (IIS-Aragón), 50009-Zaragoza, Spain.

^g^ Health Sciences Institute of Aragón (IACS), 50009-Zaragoza, Spain.

^h^ Fundación Araid, 50018- Zaragoza, Spain.

* Corresponding author

E-mail: [victorse@unizar.es](mailto:victorse@unizar.es) and mpmartind.iacs@aragon.es

**

**

**Figure S1**. Hysteresis loop measured at 300 K for the Fe_3_O_4_–EDC-NHS-NPs. The NPs show superparamagnetic properties at room temperature.


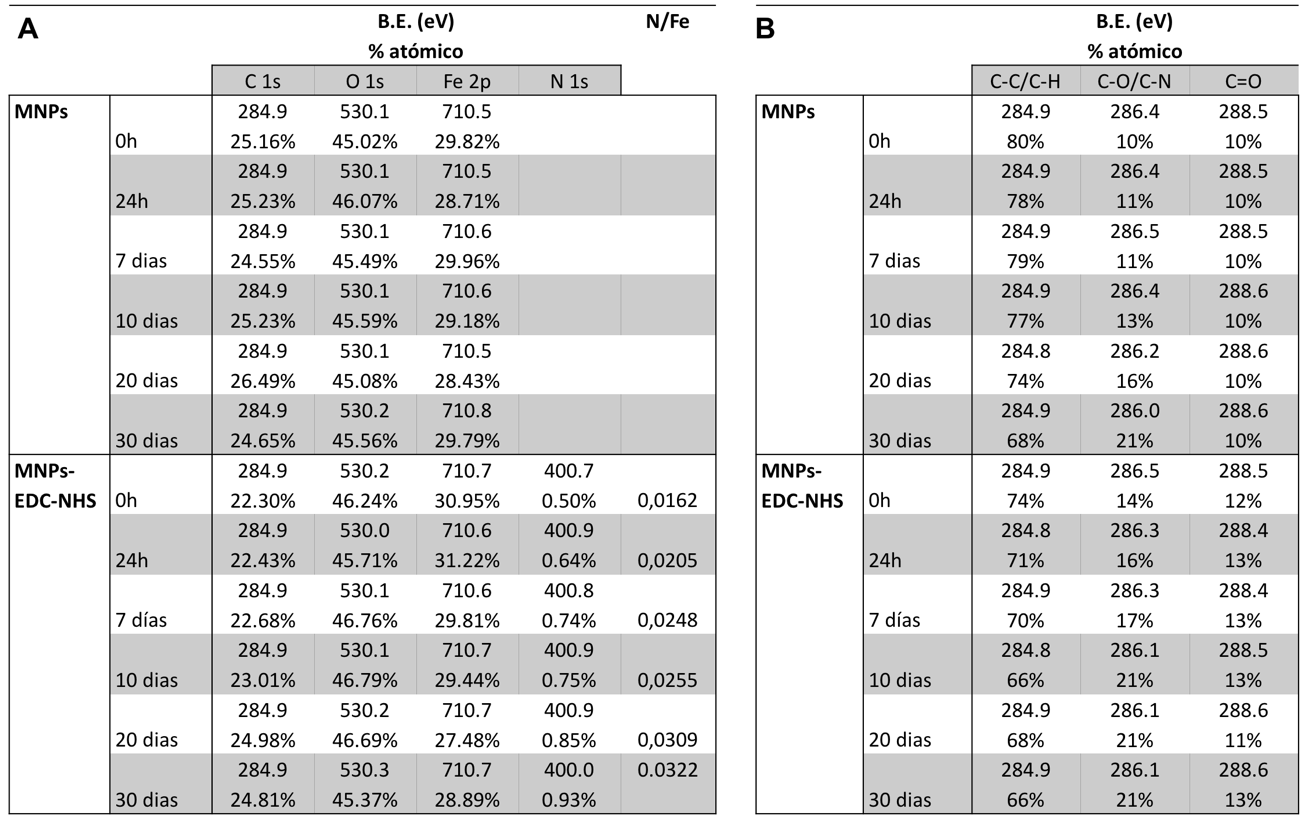
**Figure S2**. A) Complete atomic percentage of Fe_3_O_4_NPs and Fe_3_O_4_–EDC-NHS-NPs during 30 days after their synthesis. B) Study of the atomic environment of the C present in the NPs.


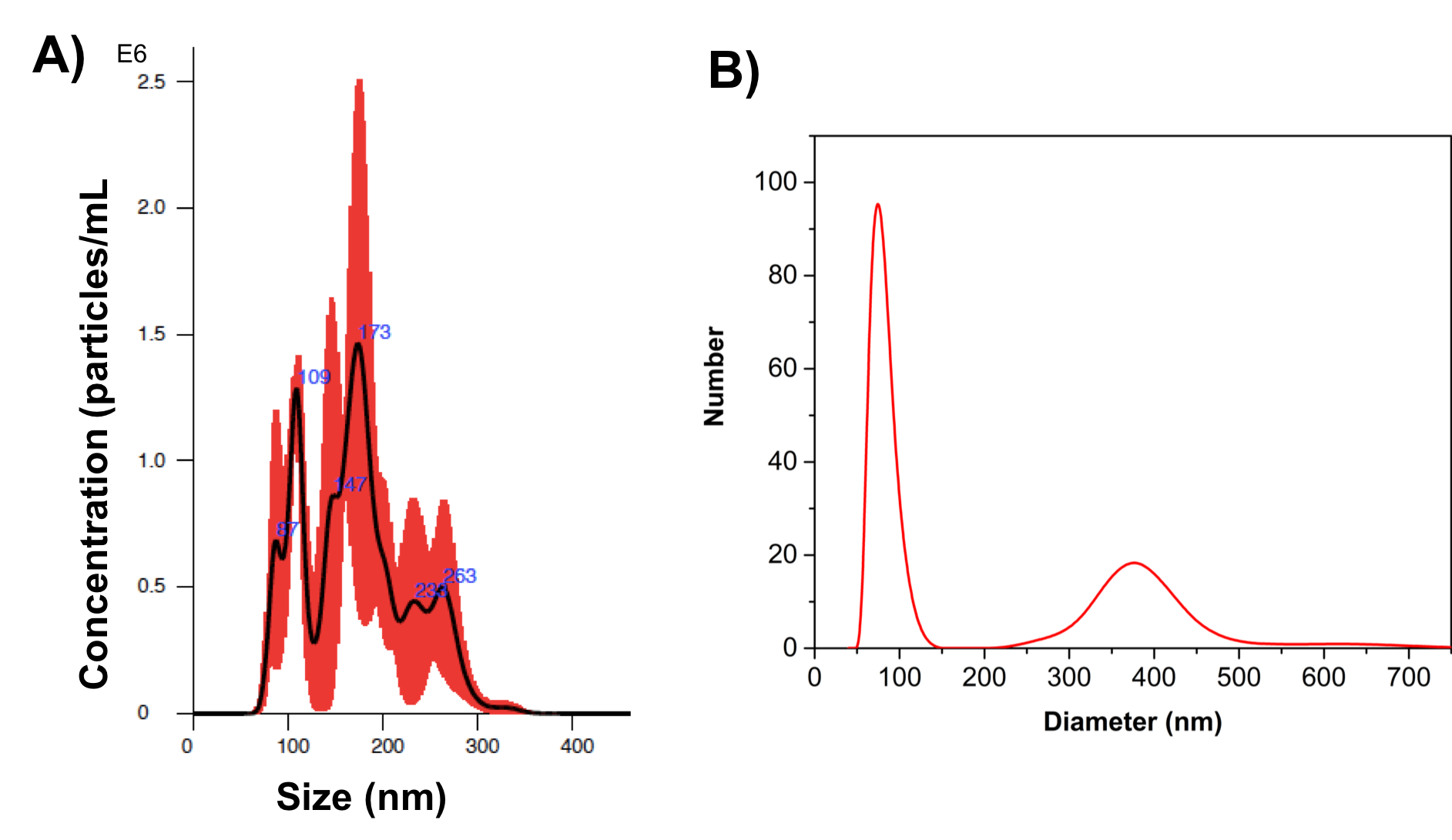


**Figure S3**. A) NTA of control exosomes and B) DLS of magnetic nanoparticles employed for the exosome capture.


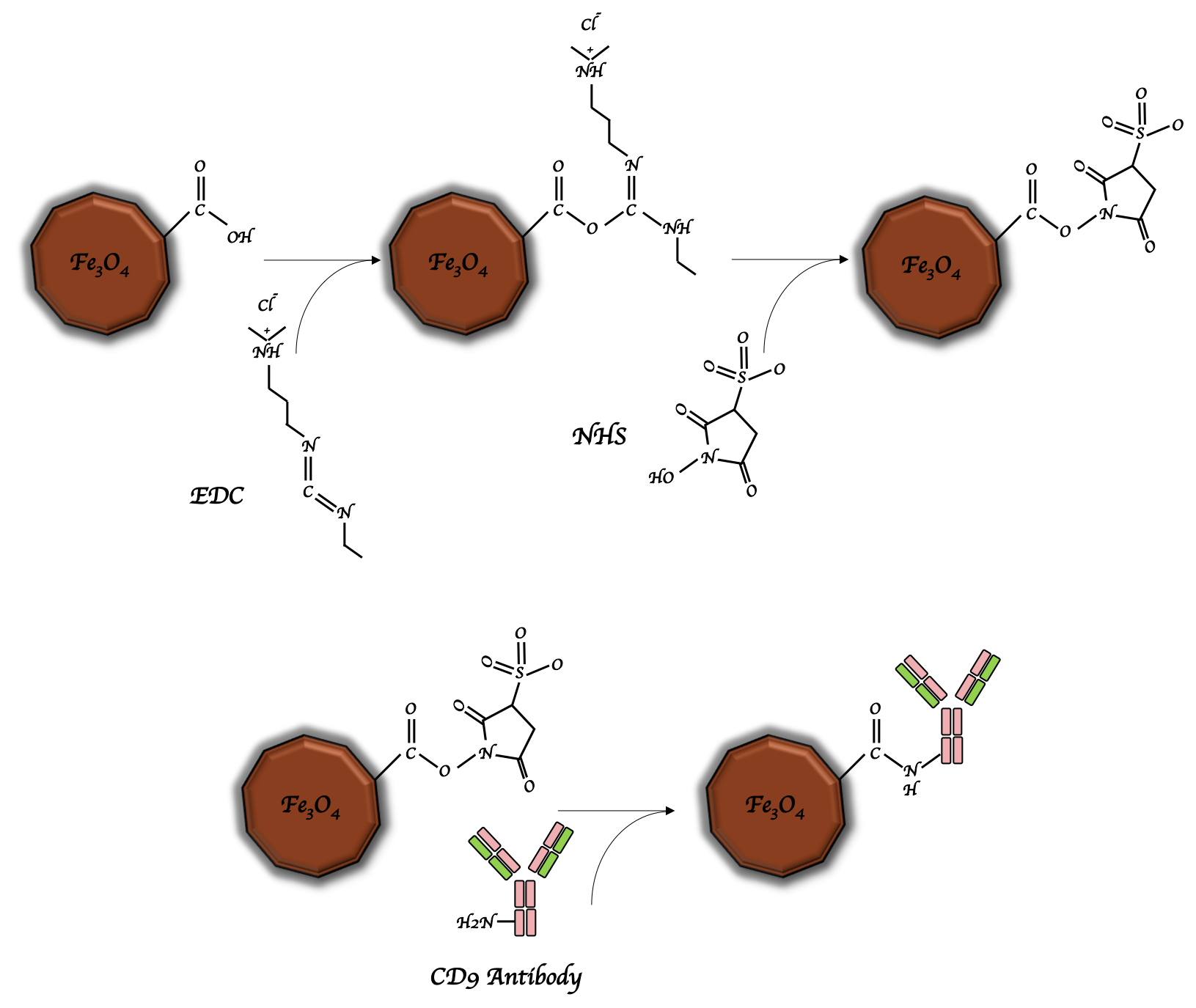


**Figure S4**. A) Scheme of the functionalization of the MNPs with the EDC-NHS linker, followed by the CD9 antibody binding.
